# TGF-βRII regulates glucose metabolism in oral cancer-associated fibroblasts via promoting PKM2 nuclear translocation

**DOI:** 10.1038/s41420-021-00804-6

**Published:** 2022-01-10

**Authors:** Fanglong Wu, Shimeng Wang, Qingxiang Zeng, Junjiang Liu, Jin Yang, Jingtian Mu, Hongdang Xu, Lanyan Wu, Qinghong Gao, Xin He, Ying Liu, Hongmei Zhou

**Affiliations:** 1 State Key Laboratory of Oral Diseases, National Center of Stomatology, National Clinical Research Center for Oral Diseases, Frontier Innovation Center for Dental Medicine Plus, West China Hospital of Stomatology, Sichuan University, 610041 Chengdu, Sichuan People’s Republic of China; 2 Department of Oral Pathology, West China Hospital of Stomatology, Sichuan University, 610041 Chengdu, Sichuan People’s Republic of China; 3 Department of Oral and Maxillofacial Surgery, West China Hospital of Stomatology, Sichuan University, 610041 Chengdu, Sichuan People’s Republic of China; 4 College & Hospital of Stomatology, Anhui Medical University, Key Laboratory of Oral Diseases Research of Anhui Province, Hefei, 230032 People’s Republic of China; 5 Department of Stomatology, North Sichuan Medical College, Affiliated Hospital of North Sichuan Medical College, Nanchong, 637000 People’s Republic of China

**Keywords:** Cancer metabolism, Tumour biomarkers

## Abstract

Cancer-associated fibroblasts (CAFs) are highly heterogeneous and differentiated stromal cells that promote tumor progression via remodeling of extracellular matrix, maintenance of stemness, angiogenesis, and modulation of tumor metabolism. Aerobic glycolysis is characterized by an increased uptake of glucose for conversion into lactate under sufficient oxygen conditions, and this metabolic process occurs at the site of energy exchange between CAFs and cancer cells. As a hallmark of cancer, metabolic reprogramming of CAFs is defined as reverse Warburg effect (RWE), characterized by increased lactate, glutamine, and pyruvate, etc. derived from aerobic glycolysis. Given that the TGF-β signal cascade plays a critical role in RWE mainly through metabolic reprogramming related proteins including pyruvate kinase muscle isozyme 2 (PKM2), however, the role of nuclear PKM2 in modifying glycolysis remains largely unknown. In this study, using a series of in vitro and in vivo experiments, we provide evidence that TGF-βRII overexpression suppresses glucose metabolism in CAFs by attenuating PKM2 nuclear translocation, thereby inhibiting oral cancer tumor growth. This study highlights a novel pathway that explains the role of TGF-βRII in CAFs glucose metabolism and suggests that targeting TGF-βRII in CAFs might represent a therapeutic approach for oral cancer.

## Introduction

As the 1st or 2nd worldwide cause of death of individuals 70 years of age and younger in 112 of 183 countries, cancer was responsible for 10.0 million deaths globally in 2020 [1]. Tumor, as an organoid, supported by tumor microenvironment (TME) consists of extracellular components and various cell types [2–4]. Among them, cancer-associated fibroblasts (CAFs), contribute to tumor progression because of their versatile roles in various tumor behaviors including in extracellular matrix remodeling, carcinogenesis, angiogenesis, maintenance of stemness, drug resistance, and modulation of tumor metabolism [5, 6]. Of note, CAF-mediated glycolytic metabolism not only provides energy but also degrades acid products in the surrounding environment to promote tumor cell survival in various cancers, such as lung [5], prostate [7], ovarian [8], and oral cancers [9].

Recently, CAF-mediated metabolic reprogramming has been described as the reverse Warburg effect (RWE) and characterized by an increased level of lactate, glutamine, fatty acids, and pyruvate derived from glycolytic metabolism [10]. In addition, the TGF-β signaling cascade plays a critical role in regulating the RWE process through the induction of metabolic reprogramming-related proteins [11, 12]. To define the role of TGF-beta receptor type-2 (TGF-βRII) in squamous cell carcinoma (SCC), several mouse models with TGF-βRII deletions have been established. For instance, Lu et al. found that TGF-βRII loss in oral keratinocytes was not an SCC initiation event [13]. In contrast, Guasch et al. provided evidence showing that K14-Cre/TGF-βRII^−/−^ mice develop spontaneous anal and genital SCC [14]. Although Lu et al. found that TGF-βRII loss in head and neck cancer was a common event [13], TGF-βRII and its role in oral CAF-mediated metabolic reprogramming have remained largely unknown during the past decade.

Pyruvate kinase M2 (PKM2) overexpression is commonly found in cancers, including gastric, esophageal, hepatocellular, and oral cancers [15, 16]. As the critical metabolic and nonmetabolic regulator, PKM2 regulates final and rate-limiting reactions in glycolysis, particularly in regulating genetic transcription [17]. To study the roles of PKM2 in oral CAFs, we performed a systematic biology analysis and found that PKM2 was closely related to TGF-βRII. Mechanistically, overexpressed TGF-βRII suppressed glucose metabolism in oral CAFs by attenuating PKM2 nuclear translocation. Our study paves the way for targeting PKM2 in CAFs, representing a new potential therapeutic avenue in oral cancer.

## Results

### The relationship between TGF-βRII and glucose metabolism in human oral cancer and oral CAFs

**Fig. 1:**
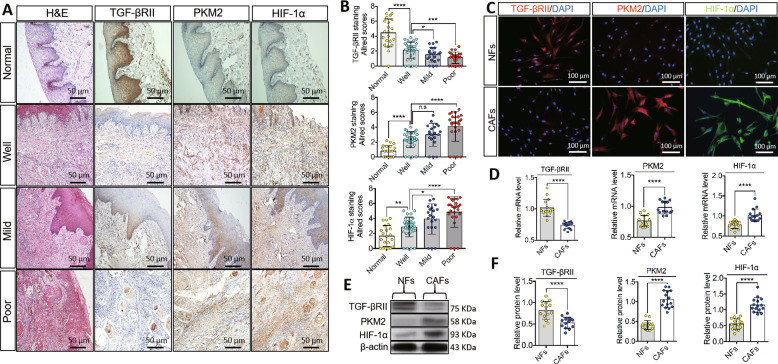
Expressions of TGF-βRII, PKM2, and HIF-1α in human oral tissues, normal fibroblasts (NFs), and oral cancer-associated fibroblasts (CAFs). **A**, **B** Positive expression of TGF-βRII, mainly located in the cellular membrane and cytoplasm, was detected in the normal tissue and it attenuated gradually in well, mildly and poorly differentiated oral squamous cell carcinomas (OSCCs) with a significant statistical difference among groups. Conversely, PKM2 and HIF-1α were weakly positive in the cytoplasm and/or nucleus in the normal tissue and exhibited with augmented positive staining and area in OSCCs. Scale bar = 50 μm. **C** Representative fluorescence images of immunostaining for TGF-βRII, PKM2, and HIF-1α were detected in NFs and CAFs with DAPI positive nuclei. Scale bar = 100 μm. **D** Quantification of TGF-βRII, PKM2, and HIF-1α mRNA levels in NFs and CAFs exhibited a significant statistical difference. **E** Immunoblot showed a reduction of TGF-βRII in CAFs but PKM2 and HIF-1α increased in CAFs when compared to NFs. **F** Quantification of TGF-βRII, PKM2, and HIF-1α protein levels in NFs and CAFs with a significant statistical difference. Relative mRNA or protein expression level was quantified after normalization to β-actin. *n* ≥ 15; error bars, mean ± SD; n.s not significant, *P < 0.05, **P < 0.01, ***P < 0.001, ****P < 0.0001; *t* test.

We enrolled 106 patients, including 85 patients with OSCCs (Table 1) and 21 who underwent maxillofacial plastic surgery or tooth extraction. We detected TGF-βRII, PKM2, and HIF-1α expression (Fig. 1). We found that TGF-βRII expression was decreased in normal tissues, well-differentiated OSCC tissues, mild-to-poorly differentiated OSCC tissues, whereas PKM2 and HIF-1α expression exhibited the opposite patterns (Fig. 1A). Based on Allred scores [18], these differences were statistically significant compared to normal tissues and tissues in different clinicopathologic stages (Fig. 1B).

**Table 1:** Clinical histopathological features of OSCC samples used for IHC staining, CAFs’ isolation, culture and transfection.

Factors	Subtypes	*n*_1_ (*n*_1_/*N*_1_)	*n*_2_ (*n*_2_/*N*_2_)	*n*_3_ (*n*_3_/*N*_3_)
Gender	Male	55 (64.7%)	9 (60.0%)	3 (50.0%)
	Female	30 (35.3%)	6 (40.0%)	3 (50.0%)
Age (years)	≤60	43 (50.6%)	7 (46.7%)	5 (83.3%)
	>60	42 (49.4%)	8 (53.3%)	1 (16.7%)
Location in the oral site	Tongue	17 (20.0%)	4 (26.7%)	2 (33.3%)
	Buccal	19 (22.4%)	6 (40.0%)	1 (16.7%)
	Gingival	7 (8.2%)	2 (13.3%)	1 (16.7%)
	Floor of oral	12 (14.1%)	2 (13.3%)	2 (33.3%)
	Others	30 (35.3%)	1 (6.7%)	0 (0.0%)
Differentiation	Well	37 (43.5%)	6 (40.0%)	3 (50.0%)
	Mild	23 (27.1%)	5 (33.3%)	2 (33.3%)
	Poor	25 (29.4%)	4 (26.7%)	1 (16.7%)
T stage (tumor volume)	T1	21 (24.7%)	4 (26.7%)	3 (50.0%)
	T2	34 (40.0%)	6 (40%)	2 (33.3%)
	T3	18 (21.2%)	1 (6.7%)	0 (0.0%)
	T4	12 (14.1%)	4 (26.7%)	1 (16.7%)
Lymph node metastasis	Negative	46 (54.1%)	8 (53.3%)	3 (50.0%)
	Positive	39 (45.9%)	7 (46.7%)	3 (50.0%)
Clinical stage	I/II	57 (67.1%)	7 (46.7%)	3 (50.0%)
	III/IV	28 (32.9%)	8 (53.3%)	3 (50.0%)

N/T number in total cases, *n*_1_ number of OSCC tissues for IHC staining, *N*_1_ 85 cases, *n*_2_ number of OSCC tissues for CAFs’ isolation and primary cell culture, *N*_2_ 15 cases, *n*_3_ number of OSCC tissues for CAFs’ transfection in vitro, *N*_3_ 6 cases.

Using immunocytochemistry (ICC), CAFs, and NFs were firstly identified with the differential expression levels of cytokeratin, vimentin, α-smooth muscle actin, fibroblast activated protein, fibroblast specific protein-1, and platelet-derived growth factor receptor β (PDGFR-β) in CAFs and NFs, respectively (Fig. S2). To explore the expressions TGF-βRII, PKM2, and HIF-1α, we performed immunofluorescence (IF) staining. Similarly, we found that TGF-βRII expression was attenuated in NFs, whereas PKM2 and HIF-1α expression was upregulated in CAFs (Fig. 1C). TGF-βRII protein and mRNA levels were increased in NFs compared with CAFs, whereas glucose metabolism-related PKM2 and HIF-1α levels were lower in NFs compared with CAFs (Fig. 1D–F).

### TGF-βRII overexpression inhibited the glycolysis in CAFs and suppressed tumor growth in vivo

**Fig. 2:**
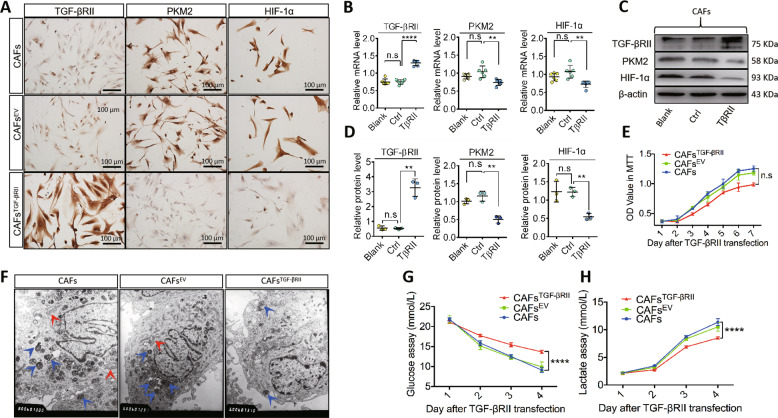
Overexpressed TGF-βRII inhibited the glycolysis in CAFs and suppressed tumor growth. **A** By immunocyte chemistry staining, TGF-βRII overexpressed in the intervened CAFs, mainly located in the cellular membrane and cytoplasm, while PKM2 and HIF-1α were weakly positive in the controls. **B**–**D** Immunoblot showed a reduction of PKM2 and HIF-1α when TGF-βRII was augmented in CAFs. Quantification of TGF-βRII, PKM2, and HIF-1α mRNA or protein levels in CAFs with a significant statistical difference among the groups. **E** There was no significant statistic between the CAFs with overexpressed TGF-βRII and the controls by MTT assay. **F** Abundant autophagosomes and mitochondria were observed in the controls while less in the CAFs with overexpressed TGF-βRII under the electron microscope (blue arrows: autophagosomes; red arrows: mitochondria). **G** Compared to controls, by glucose assay, upregulated TGF-βRII attenuated the glucose uptake in the CAFs. **H** Less production of lactic acid was detected in the experimental CAFs than the controls with a significant statistical difference. Relative mRNA or protein expression level was quantified after normalization to β-actin. Scale bar = 100 μm. *n* ≥ 3; error bars, mean ± SD; n.s. not significant, **P < 0.01, ***P < 0.001, ****P < 0.0001; *t* test.

Increased TGF-βRII expression correlates with PKM2 and HIF-1α, so it is possible that TGF-βRII might regulate glycolysis in oral CAFs, leading to affect the tumor progression in OSCC. To investigate the role of TGF-βRII, lentivirus transfection was performed in the present study (Fig. S3). First, using ICC, we confirmed that TGF-βRII was upregulated upon lentivirus transfection: however, PKM2 and HIF-1α were downregulated in CAFs transfected with lentivirus vector (Fig. 2A). Moreover, decreased PKM2 and HIF-1α protein and mRNA expression were induced by TGF-βRII overexpression (Fig. 2B–D). To explore the effects of TGF-βRII overexpression on cell behaviors in oral CAFs, we performed MTT assays to evaluate cell proliferation. We found that although the cell proliferation of CAFs transfected with lentivirus was slightly lower than that of the controls, no significant difference was noted (Fig. 2E).

**Fig. 3:**
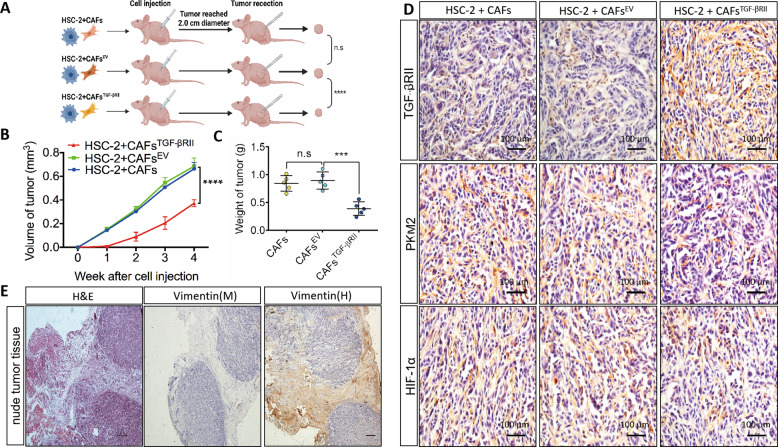
Overexpressed TGF-βRII suppressed tumor growth. **A** Schematic diagram of xenograft mouse models in vivo. **B**, **C** HSC-2 tumors were measured with calipers, and volume was calculated every week; the difference between controls and experimental groups of tumor volume and weight were found at the final time point. **D** Representative immunohistochemistry staining of TGF-βRII, PKM2, and HIF-1α in tumors of HSC-2-bearing nude mice. **E** Verification of the source of transplanted tumor cells. The staining of anti-mouse vimentin was negative while anti-human vimentin was positive. *n* ≥ 3; error bars, mean ± SD; n.s. not significant, **P < 0.01, ***P < 0.001, ****P < 0.0001; *t* test.

In the context of glycolysis, many mitochondria, autophagosomes, and actin microfilaments were observed by electron microscopy in controls, whereas fewer autophagosomes and actin microfilaments were noted in CAFs^TGF-βRII^ (Fig. 2F). Importantly, we provided evidence that TGF-βRII overexpression decreases glucose uptake and lactate secretion (Fig. 2G, H). To further identify the role of TGF-βRII overexpression in oral cancer, we generated xenograft mouse models in vivo (Fig. 3A). Our data showed that both tumor volume and tumor weight decreased in HSC-2 + CAFs^TGF-βRII^ cells compared with the controls (Fig. 3B, C). Staining also revealed that PKM2 and HIF-1α expression decreased in HSC-2 + CAFs^TGF-βRII^ cells (Fig. 3D). To further address if human mesenchymal cells could be replaced by recipient mouse fibroblasts, we performed anti-mouse and human vimentin antibodies respectively and found that anti-mouse vimentin was negative while positive of anti-human vimentin in tumor tissue (Fig. 3E), demonstrating that human mesenchymal cells were not replaced by recipient mouse fibroblasts in this mouse model.

### PKM2 acts as a key regulator of TGF-βRII-mediated glycolysis in oral CAFs

**Fig. 4:**
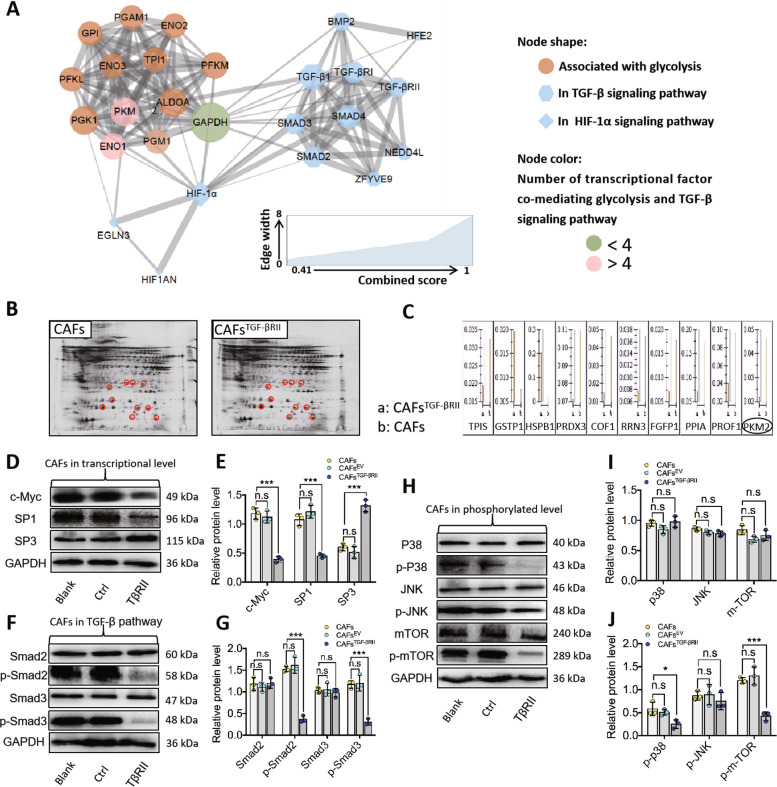
PKM2 acted as a key regulator in TGF-βRII mediating glycolysis in oral CAFs. **A** Cytoscape visualization of the protein network in glycolysis with combined analysis of TGF-β and HIF-1α signaling pathways. Protein–protein interaction network and transcriptional factor co-mediating glycolysis and TGF-β signaling pathway were generated by String and visualized by Cytoscape. PKM2 exhibited a closer relationship with the TGF-β signaling pathway than HIF-1α in this network analysis. **B**, **C** By 2-dimensional electrophoresis (2-DE) staining in proteomics analysis of glycose metabolism, PKM2 was downregulated by the overexpressed TGF-βRII in CAFs (fold change of PKM2 was 2.13021). **D**, **E** By western blot (WB), c-Myc, and SP1 were decreased by upregulation of TGF-βRII while SP3 was increased in CAFs^TGF-βRII^ when compared to CAFs^EV^ and CAFs, respectively. **F**, **G** Immunoblot showed a reduction of phosphorylated Smad2/3 in CAFs^TGF-βRII^ while no obvious change of total Smad2/3. **H**–**J** Immunoblot showed a reduction of phosphorylated P38 and mTOR, however, no obvious difference of total P38, JNK, mTOR, and p-JNK among the groups was observed. The relative protein expression level was quantified after normalization to GAPDH. *n* = 3 independent experiments; error bars, mean ± SD; n.s. not significant, *P < 0.05, ***P < 0.001; *t* test.

Human PPI analysis revealed that there were 10 proteins related to glycolysis including GAPDH, phosphoglycerate mutase 1 (PGAM1), Aldolase A (ALDOA), PKM2, phosphoglycerate kinase 1 (PGK1), phosphofructokinase, liver type (PFKL), glucose-6-phosphate isomerase (GPI), enolase 1 (ENO1), enolase 2 (ENO2), and phosphofructokinase, muscle (PFKM,) exhibit a close relationship with the TGF-β signaling pathway in the human protein reference database (HPRD) (Fig. 4A). Furthermore, as shown in Fig. 4A, we calculated the number of transcriptional regulatory factors involved in the TGF-β signaling pathway and glycolysis proteins in the human transcriptional regulation interactions database (HTRIdb). We found that the top ten glycolysis proteins related to the TGF-β signaling pathway included ENO1, hexokinase 2 (HK2), hexokinase 1 (HK1), PKM2, bisphosphoglycerate mutase (BPGM), aldo-keto reductase family 1 member A1 (AKR1A1), phosphoenolpyruvate carboxykinase 2 (PCK2), galactose mutarotase (GALM), lactate dehydrogenase B (LDHB), and aldehyde dehydrogenase 1 family member A3 (ALDH1A3) (Fig. 4A).

Given that a systems biology strategy that combines experimental and computational analyses for prediction should focus on the most stable protein, we selected PKM2 in combination with the TGF-β signaling pathway. Importantly, to further explore whether PKM2 is located downstream of the TGF-βRII-mediated signaling pathway, we performed the 2-DE staining to investigate whether TGF-βRII overexpression regulates the proteomics of glucose metabolism and found that PKM2 was downregulated in oral CAFs (Fig. 4B, C) (Table 2).

**Table 2:** Analysis of proteomics related to the glucose metabolism in CAFs with overexpressed TGF-βRII.

Accession no.	Protein name	Protein MW	Protein PI	Pep. count	Protein score	Change fold
PRDX3	Thioredoxin-dependent peroxide reductase	28017.3	7.67	4	224	−1.52799
HSPB1	Heat shock protein beta-1	122825.5	5.98	9	578	2.60578
GSTP1	GlutathioneS-transferase	23569.1	5.43	7	344	1.5379
TPIS	Triosephosphate isomerase	31056.8	5.65	16	717	−1.66946
COF1	Cofilin-1	18718.7	8.22	10	350	−3.15328
RRN3	RNA polymerase I-specific transcription initiation factor	74858.1	5.4	10	45	−2.15407
PPIA	Peptidyl-prolyl cis-trans isomerase A	18,229	7.68	10	609	−2.18749
PROF1	Profilin-1	15215.6	8.44	12	472	−2.42656
FGFP1	Fibroblast growth factor- binding protein	26874.7	9.28	8	41	−2.44656
PKM2	Pyruvate kinase muscle isozyme 2	58,470	7.95	21	69	−2.13021

Fold change (CAFs vs. CAFs^TGF-βRII^) > 1.5.

TGF-βRII regulates PKM2 expression, as shown in Fig. 4D, E. We also found that c-Myc and Sp1 transcription factor (SP1) were downregulated, whereas SP3 was upregulated significantly. Furthermore, we explored the signaling pathways related to PKM2, including mTOR, p38, and JNK. WB data showed that Smad2 and Smad3 displayed no obvious changes, whereas p-Smad2 and p-Smad3 exhibited a decreased expression (Fig. 4F, G). Similarly, total mTOR, p38, and JNK expression exhibited no significant change; however, mTOR and p38 phosphorylation were attenuated significantly by TGF-βRII overexpression (Fig. 4H–J). Based on these findings, we revealed that TGF-βRII overexpression potentially inhibits several signaling pathways related to PKM2 and TGF-β signaling, subsequently decreasing PKM2 expression.

### TGF-βRII Overexpression affects the cellular location of PKM2 and its nuclear function in CAFs

**Fig. 5:**
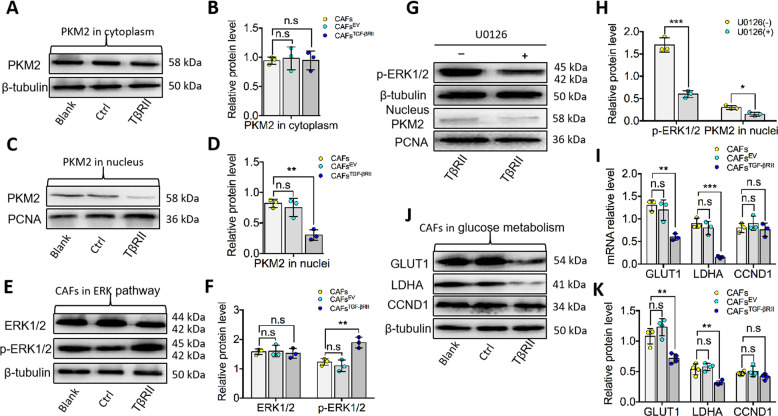
Overexpressed TGF-βRII inhibited PKM2 nuclear translocation to glycolysis. **A**, **B** In protein level, PKM2 in the cytoplasm displayed with no obvious change among the groups. **C**, **D** Overexpression of TGF-βRII decreased PKM2 level in the nucleus of CAFs^TGF-βRII^ with a significant statistical difference. **E**, **F** Immunoblot showed an augmentation of phosphorylated ERK1/2 in CAFs^TGF-βRII^ while no obvious change of non-phosphorylated ERK1/2. **G**, **H** In CAFs^TGF-βRII^ cells, the specific inhibitor of p-ERK1/2 (U0126) suppressed its phosphorylation and inhibited the PKM2 level in the nucleus simultaneously. **I**–**K** Immunoblot showed a reduction of GLUT1 and LDHA decreased in CAFs^TGF-βRII^ with a statistical difference both in the protein and mRNA level while CCND1 exhibited no obvious change. Relative mRNA or protein expression level was quantified after normalization to β-tubulin or PCNA. *n* = 3 independent experiments; error bars, mean ± SD; n.s, not significant, **P < 0.01, ***P < 0.001; *t* test.

PKM2’s function largely depends on its distribution in cells. First, we isolated PKM2 protein from the cytoplasm and the nucleus. Using WB, we observed no obvious effect on PKM2 expression in the cytoplasm among the CAFs^TGF-βRII^, CAFs^EV^, and CAFs (Fig. 5A, B). Intriguingly, compared to CAFs^EV^ and CAFs, TGF-βRII overexpression decreased PKM2 expression in the nucleus (Fig. 5C, D). Furthermore, we explored the ERK1/2 signaling pathway, which is closely related to PKM2 nuclear translocation. We provided evidence TGF-βRII overexpression does not affect the total level of ERK1/2. However, TGF-βRII overexpression activates the ERK1/2 signaling pathway by promoting ERK1/2 phosphorylation (Fig. 5E, F). To further identify whether PKM2 is located downstream of TGF-βRII and mediates the glycolysis pathway through the ERK1/2 signaling pathway, we employed an inhibitor of the ERK1/2 pathway (U0126) in vitro experiments. Our results showed that U0126 decreased pERK1/2 and PKM2 in the nuclei of CAFs^TGF-βRII^ (Fig. 5G, H).

We next explore the downstream targeted genes of PKM2. Compared with controls cells, glucose transporter 1 (GLUT1) and lactate dehydrogenase A (LDHA) expression is significantly decreased in CAFs^TGF-βRII^, whereas coding for cyclinD1 (CCND1) exhibited no obvious downregulation (Fig. 5I). At the protein level, GLUT1 and LDHA expression was decreased significantly in CAFs^TGF-βRII^ compared to CAFs^EV^ and CAFs, and no significant attenuation of CCND1 was noted after overexpressing TGF-βRII (Fig. 5J, K).

## Discussion

Stromal CAFs have been extensively studied and play important roles in tumor biological behaviors, including carcinogenesis, growth, invasion, recurrence, and metastasis, via TGF-β signal-mediated RWE [5]. In mammals, TGF-β signaling has been extensively studied and impacts diverse cellular processes, including differentiation, proliferation, migration, extracellular matrix remodeling, and apoptosis. All of these processes are potentially involved in various biological events, including embryogenesis, immunity regulation, fibrosis, wound healing, and tumor progression [4, 14]. Importantly, increased glucose in the cellular microenvironment upregulated the expression of TGF-βRI and TGF-βRII, subsequently leading to cell hypertrophy via TGF-β signaling pathway-mediated glucose metabolism [19]. These results indicate that TGF-βRII plays a crucial role in cellular glucose metabolism.

PKM2 is a key driver of aerobic glycolysis, Luo et al. revealed positive feedback between PKM2 and HIF-1α [20]. This finding was supported by the evidence indicating that the greater the degree of tumor malignancy, the more obvious the features of glycolysis and the higher the levels of PKM2 and HIF-1α expression [2, 21]. These data suggested that both PKM2 and HIF-1α play a crucial role in glycolysis in cancer. In support of this notion, we found that TGF-βRII correlated closely with glycolysis-related factors, including PKM2 and HIF-1α, in OSCCs and that TGF-βRII overexpression inhibited PKM2 and HIF-1α expression. These results demonstrate that TGF-βRII acts as one of the regulators of PKM2 and HIF-1α in oral CAFs.

With regard to biological differences, CAFs^TGF-βRII^ suppressed the glucose uptake and lactate secretion (Fig. 2G, H), so we hypothesize that TGF-βRII might suppress glycolysis uptake and lactate secretion by inhibiting the glycolysis regulators of PKM2 and HIF-1α. Fewer autophagosomes were observed in CAFs^TGF-βRII^ (Fig. 2F), indicating that mitochondria were less damaged in CAFs^TGF-βRII^. In this context, mitochondria would prefer aerobic metabolism, and less lactate would be generated. Mechanistically, increased TGF-βRII levels inhibit glycolysis in oral CAFs by suppressing the glycolysis regulators PKM2 and HIF-1α, thereby decreasing glucose uptake and lactate secretion.

We further performed a systematic analysis and found that PKM2 was worthy of close examination, and this notion was confirmed by proteomics analysis of glucose metabolism and staining in HSC-2 bearing tumors in mice (Fig. 3A–C). This finding demonstrated that PKM2 might be located downstream of the related signaling pathways involved in TGF-βRII-mediated glycolysis in oral CAFs. Similarly, PKM2 exerts a crucial role in tumor progression, and PKM2 intervention directly changes cellular glycolysis. Thus, PKM2 is regarded as a biological marker for tumor metabolism [22].

Numerous studies have shown that cellular PKM2 is regulated by various proteins, including c-Myc, SP1, and SP3 [18, 23]. C-Myc promoted gene transcript splicing into PKM2 while decreasing PKM1 expression [18]. SP1 promotes PKM2 upregulation, whereas SP3 acted as a transcriptional suppressor [24, 25]. In our study, we found that TGF-βRII overexpression in oral CAFs attenuated c-Myc and SP1 expression while upregulating SP3. These results demonstrate that increased TGF-βRII exhibits multiple effects on the regulators related to PKM2, thereby inhibiting the glycolysis at the transcriptional level. Mechanistically, the activation of several signaling pathways, including PI3K-AKT-mTOR, MAPK-p38, and MAPK-JNK, was related to PKM2 expression. Of note, studies have shown that mTOR activation and phosphorylation upregulate PKM2 [26]. Although the activation of the p38 and JNK pathways did not directly affect PKM2, p38 and JNK phosphorylation was correlated with PKM2 expression [27]. We found that overexpressed TGF-βRII significantly decreased mTOR and p38 phosphorylation but had no obvious effects on JNK compared to the controls, suggesting that TGF-βRII overexpression in oral CAFs may suppress PKM2 by inhibiting phosphorylated mTOR and p38. We further explored whether Smads were involved in the process of PKM2 regulation by TGF-βRII. Our results showed that TGF-βRII overexpression had no effect on Smad2 and Smad3, whereas p-Smad2/3 levels were attenuated, suggesting that the TGF-βRII/Smad pathway may be involved in the regulation of PKM2. In summary, TGF-βRII overexpression caused PKM2 inhibition and might be triggered by the common effects of multiple regulators and their related signaling pathways.

The functional properties of proteins depend on expression levels as well as cellular distributions. To explore how TGF-βRII overexpression affects the glycolysis regulators PKM2 in oral CAFs, we studied the location and function of PKM2. Accumulating data indicate that PKM2 possesses metabolic and nonmetabolic properties. On the one hand, PKM2 is located in the cytoplasm and is an important rate-limiting enzyme involved in the synthesis of Nicotinamide adenine dinucleotide (NADH) and acetyl-CoA by oxidation and decarboxylation of pyruvate in glycolysis. On the other hand, as a transcription regulator, PKM2 can be activated, translocated into the nucleus, and subsequently bind to DNA to regulate the expression of multiple genes [15, 28]. Importantly, the transcriptional activity of PKM2 was found to be independent of enzymatic activity [29]. This evidence indicates that the biological function of PKM2 is closely related to its location. Unexpectedly, we found TGF-βRII overexpression inhibited total PKM2 levels. No significant change in PKM2 levels was noted in the cytoplasm, but decreased PKM2 expression was noted in the nucleus. These results indicate that TGF-βRII upregulation potentially affects the nuclear functions of PKM2 as a transcription regulator. In the nucleus, PKM2 can bind with other transcription regulators, such as HIF-1α, β-catenin, and signal transducers and activators of transcription 3 (STAT3), subsequently leading to the activation of downstream gene expression [2, 30, 31]. Therefore, we proposed an alternative hypothesis that cellular energy metabolism is the basic condition for cell survival. Thus, PKM2 might initially address cellular metabolic demands. Then, excess PKM2 is transferred into the nucleus to function as a transcription regulator. When the total amount of PKM2 was suppressed by TGF-βRII overexpression, PKM2 in the cytoplasm is still required to maintain cell stability by providing essential energy, whereas nuclear PKM2 levels are downregulated. Our staining data showed increased PKM2 expression in CAFs compared with NFs. These data suggested that the additional PKM2 was probably located in the nucleus to act as a transcription regulator.

To date, several factors, such as IL-3 stimulation and ultraviolet radiation, are closely related to the transport of cytoplasmic PKM2 into the nucleus [29, 32]. Mechanistically, the epidermal growth factor could activate the extracellular regulated ERK1/2 signaling pathway. Then, p-ERK1/2 activates PKM2 directly, subsequently resulting in the translocation of PKM2 into the nucleus [33]. In our study, we found that TGF-βRII overexpression augmented p-ERK1/2 expression. Although U0126 significantly inhibited p-ERK1/2, it did not increase PKM2 levels. Conversely, U0126 reduced PKM2 levels in the nucleus, indicating that U0126 might attenuate the translocation of PKM2 into the nucleus induced by TGF-βRII upregulation. Based on these results, similar to PKM2 in tumor cells, the activation of ERK1/2 might promote PKM2 translocation into the nucleus in oral CAFs. Similar to that noted in a previous study, these data also supported the notion the ERK1/2 signaling pathway plays a vital role in promoting PKM2 nuclear translocation. Accordingly, TGF-βRII overexpression activated the ERK1/2 signaling pathway. As a compensation mechanism, a reduction in the total amount of PKM2 levels in cells caused a decrease in the nuclear PKM2.

**Fig. 6:**
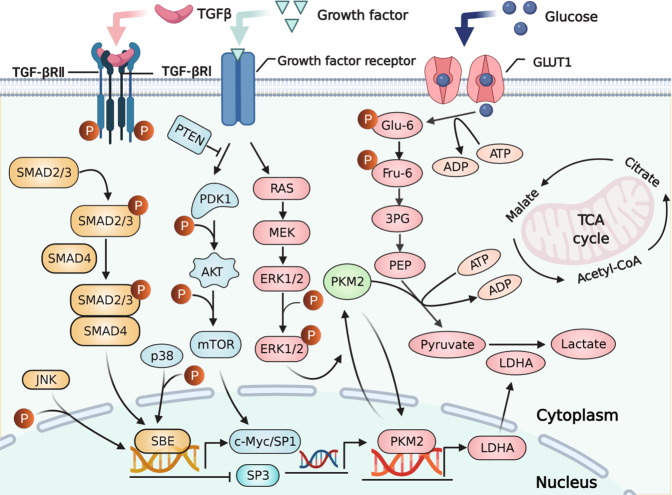
Schematic overview of TGF-βRII mediating glycolysis via PKM2 nuclear translocation in oral CAFs. TGF-βRII overexpression suppressed glucose metabolism in CAFs by attenuating PKM2 nuclear translocation, and this was triggered by multiple regulators and the related signaling pathways. TGF-βRII overexpression attenuated p-Smad2/3, augmented p-ERK1/2 expression, subsequently might promote PKM2 translocation into the nucleus in oral CAFs. Overexpressing TGF-βRII also downregulated the expression of PKM2 and GLUT1 to reduce the glucose uptake into the nucleus.

Recently, identified genes induced by PKM2, include the proto-oncogenes, such as c-Myc and CCND1, and glucose-metabolizing enzymes, such as GLUT1 and LDHA [34]. Indeed, a positive feedback loop between c-Myc and PKM2 is noted. On the one hand, c-Myc promotes the alternative splicing of PKM mRNA to promote PKM2 expression via hnRNPA1, hnRNPA2, and polypyrimidine tract binding protein. On the other hand, as a cotranscription regulator, PKM2 could increase c-Myc transcription through a feedback loop [27]. Our results showed that in the context of TGF-βRII overexpression, the activation of c-Myc transcription was decreased by PKM2 in the nucleus, suggesting that TGF-βRII overexpression in oral CAFs might suppress the positive feedback loop between the c-Myc and PKM2 and decrease their expression. In addition, we found that TGF-βRII overexpression could downregulate the expression of PKM2 and GLUT1, thereby leading to reduce glucose uptake into the nucleus in oral CAFs. As the targeted gene of PKM2, LDHA catalyzes pyruvic acid to lactic acid and generates excessive lactic acid via hyperactivation in tumor cells. In support of this notion, we also found that increased expression of TGF-βRII in oral CAFs reduced PKM2 and LDHA expression, indicating that TGF-βRII overexpression suppresses glycolysis via PKM2. Figure 6 was the schematic overview of the potential mechanism of TGF-βRII in oral CAFs mediating glycolysis via PKM2.

In conclusion, TGF-βRII overexpression suppressed glucose metabolism and tumor growth, and PKM2 nuclear translocation was involved in the TGF-βRII-mediated glycolysis in oral CAFs. Our study highlights that targeting TGF-βRII in oral CAFs exhibits potential as a therapeutic approach for oral cancer.

## Materials and methods

### Clinical tissue samples

Eighty-five oral SCC (OSCC) specimens, 21 normal oral mucosa tissues, and 15 primary human cell lines of oral CAFs and normal fibroblasts (NFs) were included. Tumor samples and oral CAFs were obtained from patients who underwent surgical resection, whereas normal tissues and NFs were obtained from normal individuals who received craniofacial plastic surgery or tooth extractions. All these samples were obtained from the West China Hospital of Stomatology at Sichuan University. This study was permitted by the Human Research Ethics Committee of West China Hospital of Stomatology at Sichuan University (No. WCHSIRB-D-2016-071).

### Primary cell culture and culture condition

Primary cell lines were generated from the tumor tissues of patients with OSCC. Briefly, the tissues were cut into small pieces (approximately 1 mm^3^) and incubated at 37 °C in a humidified atmosphere with 5% CO_2_. CAFs were obtained using the curettage method combined with trypsinization and maintained in Dulbecco’s modified Eagle’s medium (DMEM) (Gibco, Grand Island, NY, USA). Tumor cell suspensions were prepared as described, and CAFs isolation and identification were performed as described [35, 36]. In addition, CAFs from were passages 3–6 were used in the following experiments.

### Establishment of xenograft mouse models

Twenty-four nude mice (12 males and 12 females) were purchased from the State Key Laboratory of Biotherapy and Cancer Center at Sichuan University. Mice were randomly divided into four groups. All animal experiments were performed following protocols approved by the U.S. Public Health Service’s policy on the humane care and use of laboratory animals. Tumor cells and CAFs (2.0 × 10^3^) were suspended in 40 µl with 50% PBS and 50% Matrigel and injected into nude mice to form xenografts. All the cells were injected subcutaneously into the flanks of nude mice. After euthanasia, tumors were harvested when they reached 2.0 cm in diameter.

### Histology and immunostaining

The paraffin-embedded tissues were cut into 6-μm thick sections used for Hematoxylin-eosin (H&E) staining or immunostaining procedures. Antibodies against PKM2, TGF-βRII, and HIF-1α were used. Tumors were diagnosed and scored based on histology analysis by a pathologist. Scoring was based upon the number of positive cells stained in an overall assessment of all tumor tissue available. Centrosome IF images were captured using confocal microscopy with a Leica SP2 confocal microscope (Leica Microsystems Inc.).

### Quantitative real-time PCR (qRT-PCR)

For gene detection, total RNA was isolated from cells using TRIzol reagent (Invitrogen, Grand Island, NY, USA) and quantified by measuring the absorbance at 260 nm. First-strand cDNA was synthesized from total RNA using the Perfect Real-Time PrimeScript^®^ RT reagent kit (Takara, Shiga, Japan). cDNA was quantified by polymerase chain reaction (PCR) on a 7300HT Real-Time PCR System using SYBR^®^ Premix Ex TaqTM II (Takara, Shiga, Japan). GAPDH served as the endogenous control. The following PCR primer pairs were synthesized: TGF-βRII sense: 5′-GCGCTACATCGACAGCAAAG-3′ and antisense: 5′-CAATAGGCCGCATCCAAAGC-3′; PKM2 sense: 5′- AGGGAGCCACTCTCAAAATCAC-3′ and antisense: 5′-CACCTTTCTGCTTCACCTGGA-3′ (Fig. S1); HIF-1α sense: 5′-GCCGCTGGAGACACAATCATA-3′ and antisense: 5′-GGTGAGGGGAGCATTACATCAT-3′. In addition, the comparative CT method (ΔΔ CT method) was used to quantify target gene expression compared with the control.

### Western blot (WB)

Whole-cell protein extracts were prepared using lysis buffer (Roche Diagnostics, 17609500) containing phosphatase inhibitor (Roche Diagnostics, 04906837001) and protease inhibitor (Roche Diagnostics, 62032301). Proteins were subjected to 10% sodium dodecyl sulphate-polyacrylamide gel electrophoresis (SDS-PAGE) (Bio–Rad, 456-1086) using standard techniques. After transfer to the PVDF membrane (Bio-Rad, 1620177), the protein contents were probed with the following antibodies: anti-TGF-βRII (Abcam, ab259360, 1:1000), PKM2 (Abcam, ab150377, 1:10,000), HIF-1α (Abcam, ab179483, 1:1000), c-Myc (Abcam, ab32072, 1:1000), SP1 (Abcam, ab124804, 1:5000), SP3 (Abcam, ab227856, 1:3000), mTOR (Abcam, ab134903, 1:10,000), p38 (CST, #8690, 1:1000), JNK (CST, #9252,1:1000), p-mTOR (Abcam, ab109268, 1:10,000), p-p38 (CST, #4511, 1:1000), p-JNK (CST, #9255, 1:2000), Smad2 (Abcam, ab40855, 1:10,000), Smad3 (Abcam, ab40854, 1:10,000), p-Smad2 (Abcam, ab280888, 1:1000), p-Smad3 (Abcam, ab52903, 1:2000), ERK1/2 (CST, #4695, 1:1000), p-ERK1/2 (CST, #8544, 1:1000), GLUT1 (Abcam, ab115730, 1:100,000), LDHA (Abcam, ab52488, 1:10,000), and CCND1 (Abcam, ab16663, 1:200). GAPDH (Santa Cruz, sc-25778, 1:2000) and β-actin (Santa Cruz, sc-47778, 1:1000) served as controls. Horseradish peroxidase conjugated secondary antibodies were employed. The proteins were visualized using an electrochemiluminescence kit obtained from PerkinElmer (Millipore Inc., Darmstadt, Germany).

### Two-dimensional gel electrophoresis (2-DE)

First dimension-iso-electric focusing (1D-IEF): using the Protean IEF cell (Bio-Rad), IEF was performed on 7 cm IPG strips under isoelectric focusing (500 V for 1 h). After isoelectric focusing, the IPG strips were quickly placed in 2 ml of equilibration buffer A and 2 ml of equilibration buffer B for 15 min. Second dimension SDS-PAGE (2D-SDS-PAGE): IPG strips were subjected to electrophoresis after equilibration, by placement on acrylamide gels (12.5%) and casting in glass plates. The gel was transferred using an electrophoresis tank (15 mA/gel for 30 min).

### Gel staining

The silver nitrate staining method recommended in the “Two-dimensional Electrophoresis Experimental Manual” of GE Healthcare was used. Silver staining method: fixation (1 h), sensitization (1 h), washing (2 h), silver staining (45 min), washing (3 min/time, 3 times) color development (5 min), and termination (15 min). The silver-stained gel was imaged using a GS-710 optical density scanner, and Imagemaster (GE healthcare) was used to analyze and count the 2-D patterns in 3 repeated experiments. Thus, the protein difference points that met the statistical standards were obtained.

### Systems biology

To determine the sophisticated interactions among the cellular proteins, studies of cellular responses to mechanisms have relied heavily on the use of a protein–protein interaction (PPI) network system, and the prediction of PPI sites is essential for PPI networks. Computational and systematic biological analyses based on the biomolecular networks were conducted to explore the relationship between the TGF-β signaling pathway and targeted proteins related to glycolysis. Both the HTRIdb and HPRD were used to predict the key regulator at the transcriptional level and PPI level in TGF-βRII-mediated glycolysis in oral CAFs.

### Statistical analysis

All statistical analyses were performed using GraphPad Prism software (GraphPad). For all statistical analyses, if not indicated otherwise, data were analyzed by *t* test and presented as the means ± SD. The following notions were used to indicate significance: n.s. (*P* > 0.05), * (0.01 < *P* < 0.05), ** (0.001 < *P* < 0.01), *** (0.0001 < *P* < 0.001), and **** (*P* < 0.0001).

## Supplementary information

TGF-βRII regulates glucose metabolism in oral cancer-associated fibroblasts via promoting PKM2 nuclear translocation

## Data Availability

All data generated or analyzed during this study are included in this article and its supplementary information files.

## References

[1] Sung H, Ferlay J, Siegel RL, Laversanne M, Soerjomataram I, Jemal A (2021). Global Cancer Statistics 2020: GLOBOCAN estimates of incidence and mortality worldwide for 36 cancers in 185 countries. CA Cancer J Clin.

[2] Zhao H, Yang L, Baddour J, Achreja A, Bernard V, Moss T (2016). Tumor microenvironment derived exosomes pleiotropically modulate cancer cell metabolism. eLife.

[3] Wu T, Dai Y (2017). Tumor microenvironment and therapeutic response. Cancer Lett.

[4] Wu FL, Nolan K, Strait AA, Bian L, Nguyen KA, Wang JH (2019). Macrophages promote growth of squamous cancer independent of T cells. J. Dent Res.

[5] Duda P, Janczara J, McCubrey JA, Gizak A, Rakus D (2020). The reverse Warburg effect is associated with Fbp2-dependent Hif1alpha regulation in cancer cells stimulated by fibroblasts. Cells.

[6] Wu F, Yang J, Liu J, Wang Y, Mu J, Zeng Q (2021). Signaling pathways in cancer-associated fibroblasts and targeted therapy for cancer. Signal Transduct. Target Ther.

[7] Fiaschi T, Marini A, Giannoni E, Taddei ML, Gandellini P, De Donatis A (2012). Reciprocal metabolic reprogramming through lactate shuttle coordinately influences tumor-stroma interplay. Cancer Res.

[8] Radhakrishnan R, Ha JH, Jayaraman M, Liu J, Moxley KM, Isidoro C (2019). Ovarian cancer cell-derived lysophosphatidic acid induces glycolytic shift and cancer-associated fibroblast-phenotype in normal and peritumoral fibroblasts. Cancer Lett.

[9] Yang J, Shi X, Yang M, Luo J, Gao Q, Wang X (2021). Glycolysis reprogramming in cancer-associated fibroblasts promotes the growth of oral cancer through the lncRNA H19/miR-675-5p/PFKFB3 signaling pathway. Int J Oral Sci.

[10] Pavlides S, Whitaker-Menezes D, Castello-Cros R, Flomenberg N, Witkiewicz AK, Frank PG (2009). The reverse Warburg effect: aerobic glycolysis in cancer associated fibroblasts and the tumor stroma. Cell Cycle.

[11] Guido C, Whitaker-Menezes D, Capparelli C, Balliet R, Lin Z, Pestell RG (2012). Metabolic reprogramming of cancer-associated fibroblasts by TGF-beta drives tumor growth: connecting TGF-beta signaling with “Warburg-like” cancer metabolism and L-lactate production. Cell Cycle.

[12] Zhang D, Wang Y, Shi Z, Liu J, Sun P, Hou X (2015). Metabolic reprogramming of cancer-associated fibroblasts by IDH3alpha downregulation. Cell Rep.

[13] Lu SL, Herrington H, Reh D, Weber S, Bornstein S, Wang D (2006). Loss of transforming growth factor-beta type II receptor promotes metastatic head-and-neck squamous cell carcinoma. Genes Dev.

[14] Guasch G, Schober M, Pasolli HA, Conn EB, Polak L, Fuchs E (2007). Loss of TGFbeta signaling destabilizes homeostasis and promotes squamous cell carcinomas in stratified epithelia. Cancer Cell.

[15] Hsu MC, Hung WC (2018). Pyruvate kinase M2 fuels multiple aspects of cancer cells: from cellular metabolism, transcriptional regulation to extracellular signaling. Mol Cancer.

[16] Wu J, Hu L, Chen M, Cao W, Chen H, He T (2016). Pyruvate kinase M2 overexpression and poor prognosis in solid tumors of digestive system: evidence from 16 cohort studies. Onco Targets Ther.

[17] Yang W, Xia Y, Hawke D, Li X, Liang J, Xing D (2012). PKM2 phosphorylates histone H3 and promotes gene transcription and tumorigenesis. Cell.

[18] Choudhury KR, Yagle KJ, Swanson PE, Krohn KA, Rajendran JG (2010). A robust automated measure of average antibody staining in immunohistochemistry images. J Histochem Cytochem..

[19] Martinez-Outschoorn UE, Balliet RM, Rivadeneira DB, Chiavarina B, Pavlides S, Wang C (2010). Oxidative stress in cancer associated fibroblasts drives tumor-stroma co-evolution: a new paradigm for understanding tumor metabolism, the field effect and genomic instability in cancer cells. Cell Cycle.

[20] Luo W, Hu H, Chang R, Zhong J, Knabel M, O’Meally R (2011). Pyruvate kinase M2 is a PHD3-stimulated coactivator for hypoxia-inducible factor 1. Cell.

[21] Christofk HR, Vander Heiden MG, Harris MH, Ramanathan A, Gerszten RE, Wei R (2008). The M2 splice isoform of pyruvate kinase is important for cancer metabolism and tumour growth. Nature.

[22] Ouyang X, Han SN, Zhang JY, Dioletis E, Nemeth BT, Pacher P (2018). Digoxin suppresses pyruvate kinase M2-promoted HIF-1α Transactivation in Steatohepatitis. Cell Metab.

[23] Berg M, Monnin D, Cho J, Nelson L, Crits-Christoph A, Shapira M (2019). TGFβ/BMP immune signaling affects abundance and function of C. elegans gut commensals. Nat Commun..

[24] Liu T, Han C, Wang S, Fang P, Ma Z, Xu L (2019). Cancer-associated fibroblasts: an emerging target of anti-cancer immunotherapy. J Hematol.

[25] Becker LM, O’Connell JT, Vo AP, Cain MP, Tampe D, Bizarro L (2020). Epigenetic reprogramming of cancer-associated fibroblasts deregulates glucose metabolism and facilitates progression of breast cancer. Cell Rep.

[26] Wu L, Derynck R (2009). Essential role of TGF-beta signaling in glucose-induced cell hypertrophy. Dev Cell.

[27] Chappell JC, Payne LB, Rathmell WK (2019). Hypoxia, angiogenesis, and metabolism in the hereditary kidney cancers. J Clin Investig.

[28] Lv WW, Liu D, Liu XC, Feng TN, Li L, Qian BY (2018). Effects of PKM2 on global metabolic changes and prognosis in hepatocellular carcinoma: from gene expression to drug discovery. BMC Cancer.

[29] Gupta A, Ajith A, Singh S, Panday RK, Samaiya A, Shukla S (2018). PAK2-c-Myc-PKM2 axis plays an essential role in head and neck oncogenesis via regulating Warburg effect. Cell Death Dis.

[30] Yang W, Lu Z (2013). Regulation and function of pyruvate kinase M2 in cancer. Cancer Lett.

[31] Ling Z, Liu D, Zhang G, Liang Q, Xiang P, Xu Y (2017). miR-361-5p modulates metabolism and autophagy via the Sp1-mediated regulation of PKM2 in prostate cancer. Oncol Rep.

[32] Li L, Davie JR (2010). The role of Sp1 and Sp3 in normal and cancer cell biology. Ann Anat.

[33] Sun Q, Chen X, Ma J, Peng H, Wang F, Zha X (2011). Mammalian target of rapamycin up-regulation of pyruvate kinase isoenzyme type M2 is critical for aerobic glycolysis and tumor growth. Proc Natl Acad Sci USA.

[34] Wong N, Ojo D, Yan J, Tang D (2015). PKM2 contributes to cancer metabolism. Cancer Lett.

[35] Liu Y, Hu T, Shen J, Li SF, Lin JW, Zheng XH (2006). Separation, cultivation and biological characteristics of oral carcinoma-associated fibroblasts. Oral Dis.

[36] Meng W, Wu Y, He X, Liu C, Gao Q, Ge L (2014). A systems biology approach identifies effective tumor-stroma common targets for oral squamous cell carcinoma. Cancer Res.

