# Supplementary material for: TGF-βRII regulates glucose metabolism in oral cancer-associated fibroblasts via promoting PKM2 nuclear translocation
Source: Cell Death Discov. 2022 Jan 10;8:3. doi: 10.1038/s41420-021-00804-6 (PMC8748622; doi:10.1038/s41420-021-00804-6)
Supplement: Supplementary file 1 — TGF-βRII regulates glucose metabolism in oral cancer-associated fibroblasts via promoting PKM2 nuclear translocation [file 41420_2021_804_MOESM1_ESM.docx]

**TGF-βRII regulates glucose metabolism in oral cancer-associated fibroblasts via promoting PKM2 nuclear translocation**

Fanglong Wu^1＃^, Shimeng Wang^1＃^, Qingxiang Zeng^1^, Junjiang Liu^1^, Jin Yang^1^, Jingtian Mu^1^, Hongdang Xu^1^, Lanyan Wu^2^, Qinghong Gao^3^, Xin He^1,4*^, Ying Liu^1,5*^, Hongmei Zhou^1*^

Appendix Materials and Methods

**Immunocytochemistry Staining**

Primary cells were inoculated on cell slides, and immunohistochemistry assays were performed. Briefly, slides of CAFs, NFs were covered with 4% formaldehyde for 30 min at temperature and then washed with PBS three times. Then, immunocytochemistry assays were performed using a biotin-streptomycin assay kit according to the manufacturer’s protocol (Solarbio Life Science, Beijing, China). The cells were incubated with primary antibodies overnight at 4 °C and then reacted with secondary antibody. The following antibodies were used for immunostaining: Anti-α-smooth muscle actin antibody (abcam, UK, 1:200), Anti-fibroblast activated protein antibody (abcam, UK, 1:200), Anti-Vimentin antibody (Abcam, UK, 1:200), Anti-Cytokeratin antibody (abcam, UK, 1:200), Anti-Fibroblast Specific Protein-1 antibody (HUABIO, China, 1:200), Anti-Platelet derived growth factor receptor-β antibody (HUABIO, China, 1:200).


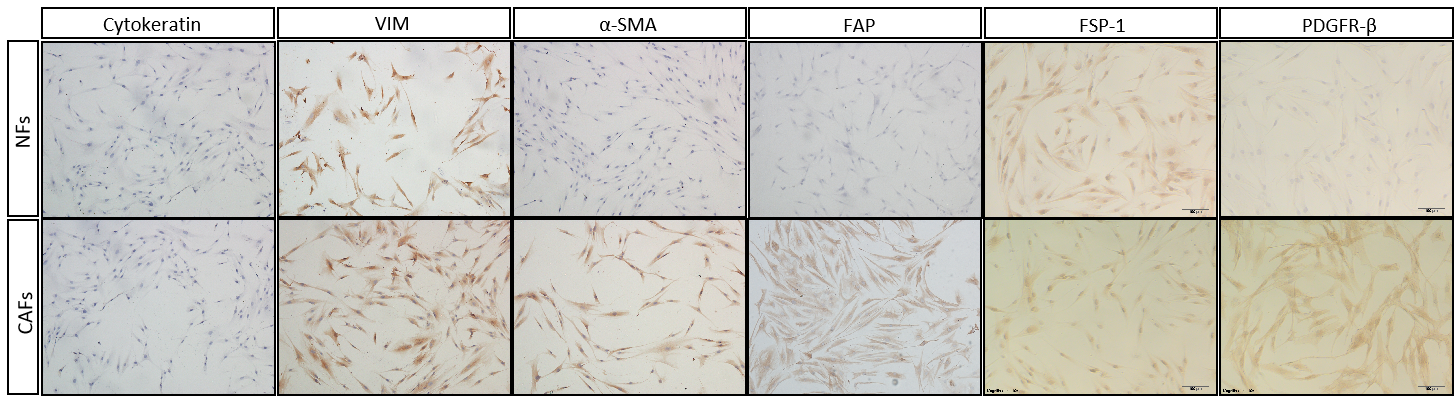


**Fig.S1.** **Immunohistochemistry cell staining of CAFs and NFs**. cytokeratin staining was negative while vimentin was positive in NFs, both α-SMA and FAP were negative in NFs. cytokeratin staining was negative while vimentin was positive in CAFs, α-SMA, FAP and FSP-1 were positive in CAFs, PDGFR-β was weak positive in NFs while strong positive in CAFs (scale bar = 100 μm).


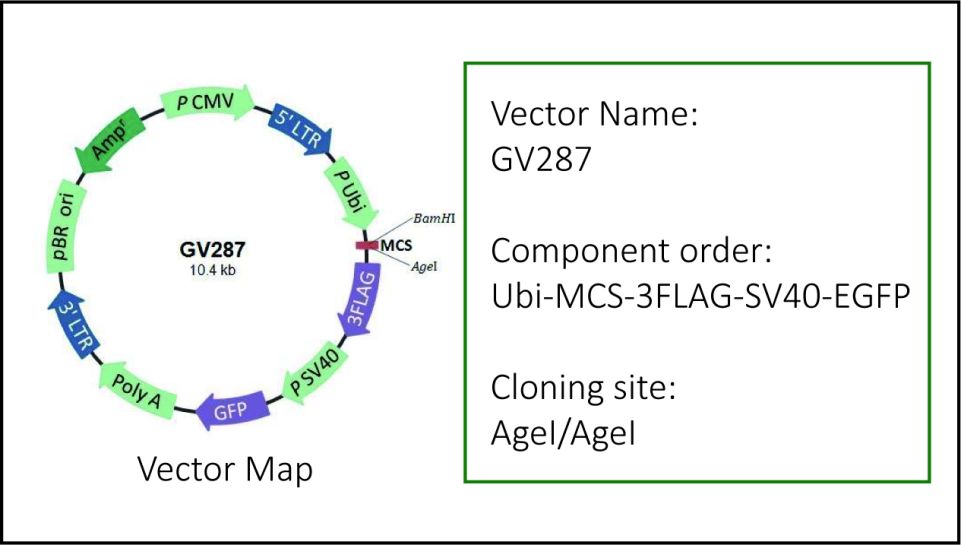


**Fig.S2. The information of the lentivirus carrying TGF-βRII gene.**


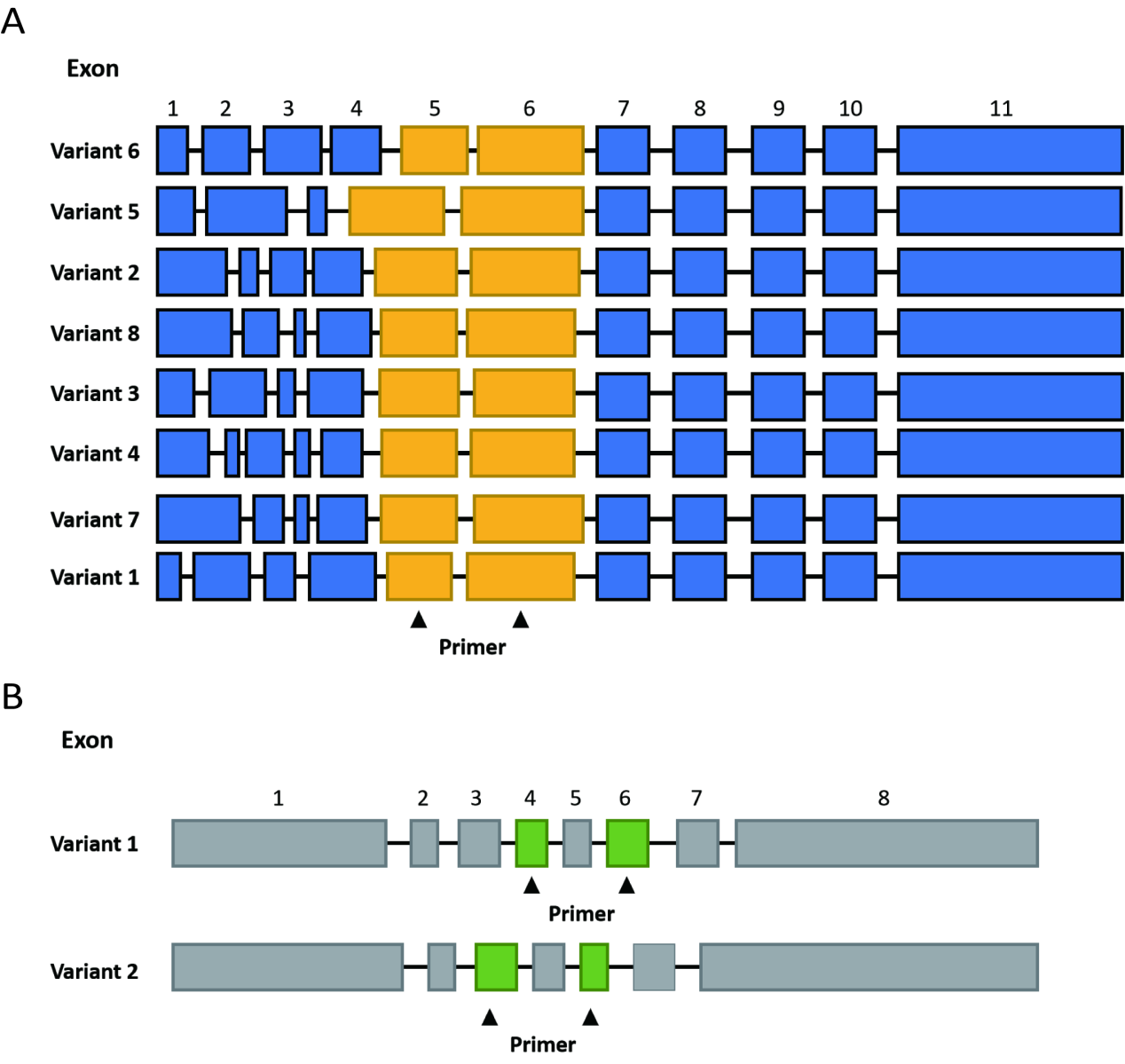


**Fig.S3. Genomic structure of PKM2 and TGF-βRII.** A. Genomic structure of PKM2 variant 6, 5, 2, 8, 3, 4, 7, and 1. Genomic information was obtained from NCBI database (V6: [NM_001206798.3](https://www.ncbi.nlm.nih.gov/entrez/viewer.fcgi?db=nucleotide&id=1890346215); V5: [NM_001206797.3](https://www.ncbi.nlm.nih.gov/entrez/viewer.fcgi?db=nucleotide&id=1890334121); V2: [NM_182470.4](https://www.ncbi.nlm.nih.gov/entrez/viewer.fcgi?db=nucleotide&id=1890274639); V8: [NM_001316318.2](https://www.ncbi.nlm.nih.gov/entrez/viewer.fcgi?db=nucleotide&id=1676318993); V3: [NM_182471.4](https://www.ncbi.nlm.nih.gov/entrez/viewer.fcgi?db=nucleotide&id=1676318636); V4: [NM_001206796.3](https://www.ncbi.nlm.nih.gov/entrez/viewer.fcgi?db=nucleotide&id=1676317989); V7: [NM_001206799.2](https://www.ncbi.nlm.nih.gov/entrez/viewer.fcgi?db=nucleotide&id=1675151011); V1: [NM_002654.6](https://www.ncbi.nlm.nih.gov/entrez/viewer.fcgi?db=nucleotide&id=1519313673)). Primers locate in common exon 5 and 6 (variant 6, 2, 8, 3, 7 and 1), in common exon 4 and 5 (variant 5), and in common exon 6 and 7 (variant 4 ). B. Genomic structure of TGF-βRII variant 1 and 2. Genomic information was obtained from NCBI database (V1: [NM_001135599.4](https://www.ncbi.nlm.nih.gov/entrez/viewer.fcgi?db=nucleotide&id=1890255549); V2: [NM_003238.6](https://www.ncbi.nlm.nih.gov/entrez/viewer.fcgi?db=nucleotide&id=1732746344)). Primers locate in common exon 4 and 6 (variant 1 ), and in common exon 3 and 5 (variant 2).
